# Effects of Quercetin Glycoside Supplementation Combined With Low-Intensity Resistance Training on Muscle Quantity and Stiffness: A Randomized, Controlled Trial

**DOI:** 10.3389/fnut.2022.912217

**Published:** 2022-07-06

**Authors:** Yuta Otsuka, Naokazu Miyamoto, Akitoshi Nagai, Takayuki Izumo, Masaaki Nakai, Masahiro Fukuda, Takuma Arimitsu, Yosuke Yamada, Takeshi Hashimoto

**Affiliations:** ^1^Institute for Health Care Science, Suntory Wellness Ltd., Kyoto, Japan; ^2^Graduate School of Health and Sports Science, Juntendo University, Chiba, Japan; ^3^Fukuda Clinic, Osaka, Japan; ^4^Faculty of Sport and Health Science, Ritsumeikan University, Kyoto, Japan; ^5^Faculty of Health Care, Undergraduate Department of Human Health, Hachinohe Gakuin University, Aomori, Japan; ^6^National Institute of Health and Nutrition, National Institutes of Biomedical Innovation, Health and Nutrition Tokyo, Tokyo, Japan

**Keywords:** muscle quality, shear wave elastography, passive muscle stiffness, whole-body lean mass, nutrition and exercise

## Abstract

**Objective:**

Aging of skeletal muscle is characterized not only by a decrease of muscle quantity but also by changes in muscle quality, such as an increase in muscle stiffness. The present study aimed to investigate the effects of supplementation with quercetin glycosides (QGs), well-known polyphenolic flavonoids, combined with resistance exercise on muscle quantity and stiffness.

**Materials and Methods:**

A randomized, controlled trial was conducted in community-dwelling, Japanese people aged 50–74 years who were randomly allocated to exercise with placebo or 200 or 500 mg of QG supplementation. All participants performed low-intensity resistance training mainly targeting thigh muscles with 40% of 1-repetition maximum, 3 days per week for 24 weeks. Muscle cross-sectional area (CSA), lean mass, and vastus lateralis (VL) muscle stiffness were measured before and after the 24-week intervention.

**Results:**

Forty-eight subjects completed the 24-week intervention. There were no significant group × time interactions in thigh CSA for primary outcome, as well as lean mass. VL muscle stiffness in the stretched position was significantly lower in both the 200 mg and 500 mg QG groups than in the placebo group after the 24-week intervention (*p* < 0.05). No significant correlation was observed between changes of VL muscle CSA and stiffness during the 24-week intervention.

**Conclusion:**

Quercetin glycoside supplementation combined with low-intensity resistance exercise improved passive muscle stiffness independently of muscle quantity.

**Clinical Trial Registration:**

[www.umin.ac.jp/ctr/], identifier [UMIN000037633].

## Introduction

Sarcopenia is characterized by an age-related decrease of skeletal muscle mass and loss of strength and/or physical function, causing high risks of multiple diseases and mortality in elderly people (1). Decreases of muscle strength and power are related not only to decreased muscle quantity but also to changes in muscle quality, such as the accumulation of intramuscular fat and/or extracellular matrix (ECM) components, with aging (2–4). In particular, an excessive accumulation of ECM components, referred to as muscle fibrosis, causes muscles to become stiff, leading to impaired muscle regeneration and functional recovery, thus decreasing the quality of life of elderly people (5, 6). The ECM accumulation is responsible for an increase in muscle stiffness, especially when the muscle is stretched (3). Recently, we have found age-related increases in muscle stiffness in the thigh in a stretched position (7). Therefore, it is crucial to simultaneously prevent not only the loss of muscle quantity but also the increase in muscle stiffness with aging.

It is well recognized that resistance training enhances muscle mass and strength to prevent the progression of sarcopenia (8). A systematic review suggested a dose-response relationship between resistance training intensity and the increase in muscle mass in elderly people (9). Our previous study also showed that a higher intensity of resistance exercise was more effective for improving both muscle quantity and quality (10). In fact, we found that low-intensity, 40% 1-repetition maximum (1-RM) exercise for 24 weeks could improve muscle quantity, but not muscle quality, although both were improved by 60% 1-RM exercise for the same duration (10). Recently, the guideline of the Asian Working Group for Sarcopenia suggested that nutritional supplementation has a supportive role in the effects of exercise on muscle mass and function (11). Therefore, even low-intensity resistance training would exert additive effects on muscle quantity and quality when combined with nutrition, whereas there have been few randomized, controlled trials that examined such combination effects (12).

Quercetin (3,5,7,3′,4′-pentahydroxyflavone) is a polyphenolic flavonoid widely distributed in plant foods such as tea, onions, and apples (13). Quercetin exists especially as glycoside forms in plants, so-called quercetin glycosides (QGs), which are enzymatically converted into the aglycone form during absorption, then exert biological actions (14). QGs are more water-soluble and bioavailable than quercetin aglycone (14), and thus they are often used as a dietary supplement for human health. There have been several studies reporting the therapeutic efficacies of quercetin, including for muscular health, such as improving muscle atrophy, through its anti-oxidative and anti-inflammatory activities (15). Quercetin administration suppressed the signaling of muscle degradation to ameliorate muscle atrophy in disuse or glucocorticoid-treated mice (16, 17). In addition, quercetin also inhibited fibrogenesis in skeletal muscle by regulating the differentiation of muscle progenitor cells (18). Thus, quercetin has the potential to improve muscle atrophy and fibrosis *in vivo*, but no clinical trials have been conducted.

The aim of the present study was to investigate the combined effect of QG supplementation and resistance training on muscle quantity and stiffness in middle-aged and elderly people. This was a 24-week, randomized, controlled trial to evaluate the effects of QG supplementation with low-intensity, 40% 1-RM, resistance training on muscle cross-sectional area (CSA), lean mass, and muscle stiffness in Japanese people aged 50–74 years. The hypothesis was that low-intensity resistance exercise combined with QG supplementation provides greater improvement in muscle quantity and stiffness than exercise alone. A further aim was to examine whether the training effects on muscle quantity and stiffness would differ depending on the dose of QGs.

## Materials and Methods

### Study Design

A randomized, double-blind, placebo-controlled, parallel-group, comparative study was designed to evaluate the effects of two different doses of QG supplementation with resistance training on muscle quantity and stiffness. Community-dwelling Japanese people living in Osaka were recruited, and 185 participants were screened. Fifty-four participants were randomly allocated to the placebo with exercise (placebo), 200 mg of QGs with exercise (low-QG), and 500 mg of QGs with exercise (high-QG) groups. Randomization was performed based on dynamic allocation to maintain balance among the groups in age, sex, and leg muscle mass using a spreadsheet program with the RAND function (Microsoft Excel 2013, Microsoft Corporation, Redmond, WA, United States). Leg muscle mass was determined by a multiple-frequency, body composition meter (MC-780A, Tanita, Tokyo, Japan; 19), as well as the sum of the muscle mass of legs and arms, which was converted to a skeletal muscle mass index (SMI) by dividing by height in meters squared (kg/m^2^). The randomization codes for both participants and groups were held in sealed opaque envelopes by two different individuals who were not engaged in the present study. A research nurse kept the envelopes closed until all data were collected and analyzed. The interventions for the 24 weeks of the present study were performed between August 2019 and March 2020. Magnetic resonance imaging (MRI), dual-energy X-ray absorptiometry (DXA), and ultrasound shear wave elastography (SWE) measurements were performed at baseline, at 12 weeks, and at 24 weeks during the intervention. Blood and urine were sampled following overnight fasting for safety assessment in the screening period, at baseline, at 12 weeks, and at 24 weeks. All participants recorded changes in physical condition and habituation during the intervention period because they were instructed not to change their lifestyles, especially exercise habits, and their compliance with the exercise intervention and capsule intake were checked regularly. The Ethics Committee of The Fukuda Clinic and Ritsumeikan University approved the study protocol in compliance with the Declaration of Helsinki. All participants provided their written, informed consent prior to their inclusion in the present study. The present study was registered in the University Hospital Medical Information Network (UMIN) Clinical Trial Registry (UMIN000037633).

### Population

The participants were community-dwelling Japanese men and women, aged 50–74 years, not engaging in exercise regularly, that is not more than twice a week and more than 30 min per times, in the past year before starting the screening. Exclusion criteria were: the presence of disease affecting the locomotor organs; the presence of cardiovascular disease limiting exercise intervention; a history of serious disorders and clinically significant systemic diseases; having problems doing the exercise intervention; planning weight loss; having previous experience with high-intensity exercise, such as bodybuilder; an irregular lifestyle; a heavy drinker or smoker; consumption of drugs or supplements that affect efficacy evaluation; consumption of drugs consecutively during interventions; not capable of undergoing MRI measurements, such as having magnetic material, a tattoo on the body, or claustrophobia; not capable of swallowing capsules; pregnant women; nursing mothers or women of child-bearing potential; and the presence of any medical condition judged by the medical investigator to be incompatible with participation in the present study.

### Outcomes

The primary outcome in the present study was the change in thigh muscle CSA on MRI over 24 weeks. Secondary outcomes were changes in vastus lateralis (VL) muscle CSA, whole-body lean mass by DXA, and shear wave velocity (SWV, an index of tissue stiffness) in three different positions by SWE. Safety was assessed based on the incidence of side effects and adverse events, such as feeling cold, lassitude, or muscular pain, among the groups during the 24-week intervention.

### Sample Size Calculations

Due to a lack of preliminary data to estimate the expected treatment difference, the sample size was set at 48 participants based on achieving a statistical power of 80% and type I error of 5% with a two-tailed test to detect a clinically meaningful difference (effect size of 0.21) in the primary outcome, setting the number of groups and measurement number of three in the repeated-measures analysis of variance (ANOVA) test. A sample size of 54 participants was required based on an expected drop-out rate of 15%.

### Measurements

#### Magnetic Resonance Imaging Measurements

To evaluate muscle CSAs, a 3.0-T MR system (MAGNETOM Skyra, Siemens Healthineers, Erlangen, Germany) was used to obtain a series of axial slices from the superior border of the patella to the greater trochanter including the rectus femoris muscle. The images of the right midthigh at the center of the end-to-end images from the 10-mm-thick slices were analyzed to measure thigh and VL muscle CSAs by SliceOmatic Ver 4.3 software (TomoVision, Magog, Canada).

#### Dual-Energy X-Ray Absorptiometry Measurements

Dual-energy X-ray absorptiometry (Lunar iDXA; GE Healthcare United Kingdom Limited, Buckinghamshire, United Kingdom) was used for whole-body composition assessment. Lean mass was obtained from leg, arm, and whole-body regions. All measurements were performed by a medical technologist of Ritsumeikan University.

#### Shear Wave Elastography Measurements

Shear wave velocity of the right VL was measured by an ultrasound SWE apparatus (Aixplorer version 12, Supersonic Imagine, Aix-en-Provence, France) with a linear array probe (SL10-2), as previously reported (7). Briefly, the ultrasound probe was placed at 50% of the thigh length and aligned in the plane of the VL fascicles so that several fascicles were identified across the B-mode image, at the following three knee joint angles: (i) the knee fully extended in the supine position; (ii) the knee flexed at 90° in the seated position; and (iii) the knee passively (supported by the examiner) fully flexed in the seated position. The SWE measurements were performed in this order. The participants were instructed to relax completely throughout the measurements. In each position, three measurements were performed (i.e., three images were acquired). The SWE data were analyzed using the software included with the ultrasound apparatus to calculate SWV over the region of interest, which was as large as possible, in VL without aponeurosis or subcutaneous adipose tissue. It was confirmed that no pixel in the region of interest reached the saturation limit of SWV (16.3 m/s). For each position, the average of three measurements was used for further analyses. All measurements and analyses of the SWE data were performed by an examiner with more than 5 years of experience.

### Resistance Exercise

Participants performed resistance exercise programs 3 days per week (every Monday, Wednesday, and Friday) for 24 weeks, as previously reported (10). Briefly, a well-trained instructor conducted the training program, which constituted a 5-min warm-up, 30-min resistance training using machines including leg extension, leg curl, leg press, and chest press, and 5-min cool-down. The training weight was 40% 1-RM, and all training was 3 sets of 14 repetitions, and the rest period between sets was 2–2.5 min. 1-RM was determined every 4 weeks to adjust the training weight for each participant, while during the first 2 weeks of the 24-week period, participants were instructed to achieve the appropriate forms with gradually increasing intensity. For evaluating 1-RM, the indirect method of the 1-RM test was used, as previously reported (10). Attendance and numbers of sets completed were checked by instructors to calculate the attendance rates.

### Experimental Supplement

Experimental supplements contained 200 mg or 500 mg of QGs. QGs were enzymatically manufactured at San-Ei Gen F.F.I., Inc. (Osaka, Japan) from isoquercitrin prepared from quercetin-3-*O*-rutinoside. Participants took six capsules including 0 mg of QGs (placebo group), 200 mg of QGs (low-QG group), or 500 mg of QGs (high-QG group) with the same color. The weights and volumes of all capsules were adjusted to be equal using dextrin, silicon oxide, and calcium stearate. Compliance with capsule intake in each participant was checked by the study diary.

### Statistical Analysis

Per-protocol set (PPS) analysis was used for efficacy assessment according to the statistical analysis plan. Baseline characteristics were compared among the groups by one-way ANOVA for quantitative variables and the chi-squared test for categorical variables. Differences between groups over time were analyzed by two-way repeated-ANOVA. When a significance of group × time interaction was observed, actual values at each measurement point and changes from baseline to 12 or 24 weeks between low-QG or high-QG and placebo groups were compared by Dunnett’s test, as well as actual values at baseline and 12 or 24 weeks in each group. Correlation analyses between muscle CSA and SWV of VL and between the sum of leg extension and leg press 1-RM (1-RM muscular strength) and SWV of VL were performed using Pearson’s correlation coefficients. The full analysis set was used for safety assessment. The incidences of side effects and adverse events were compared among groups by Bonferroni corrections after Fisher’s exact test. *P*-values < 0.05 were considered significant. Statistical analyses were carried out using IBM SPSS Statistics for Windows, Version 22.0 (IBM Corporation, Armonk, NY, United States) and JMP, Version 15.2 (SAS Institute Inc., Cary, NC, United States).

## Results

### Participant Flow and Baseline Characteristics

**FIGURE 1:**
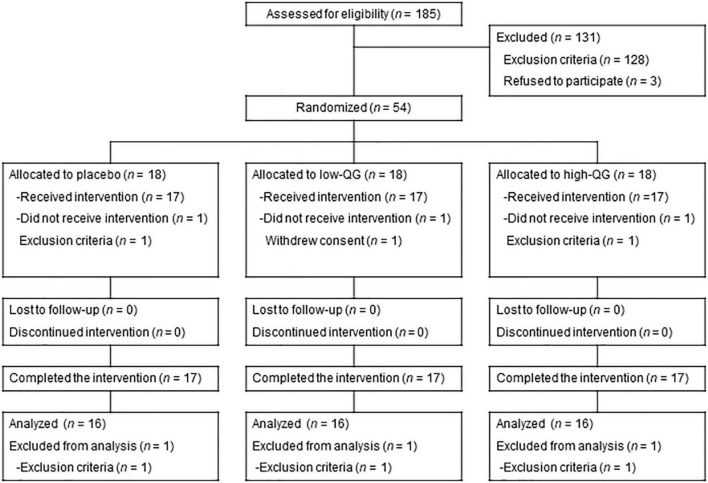
Flowchart of this study. Of the 185 participants recruited, 54 were randomly allocated to the placebo, low-QG, and high-QG groups. QG, quercetin glycoside.

Of the 54 participants allocated to the three groups, 3 withdrew consent prior to the intervention or dropped out because of the exclusion criteria. Consequently, 51 participants started and completed (*n* = 17 in the placebo group; *n* = 17 in the low-QG group; and *n* = 17 in the high-QG group) the 24-week interventions and were included in the safety assessment (full analysis set population). Another 3 participants were excluded from the analysis because they fulfilled the exclusion criteria. Therefore, 48 participants were included in the PPS population used for efficacy assessment (*n* = 16 in the placebo group; *n* = 16 in the low-QG group; *and n* = 16 in the high-QG group, see flow chart in Figure 1). Mean attendance rates during the 24-week resistance exercise program were 95.6 ± 4.7% in the placebo group, 97.0 ± 3.9% in the low-QG group, and 96.1 ± 5.5% in the high-QG group, with no significant difference among the groups (*p* = 0.718). Mean compliance rates for capsule intake during the 24-week intervention were 99.8 ± 0.6% in the placebo group, 99.5 ± 0.8% in the low-QG group, and 99.9 ± 0.3% in the high-QG group, with no significant difference among the groups (*p* = 0.235). There were no significant differences in baseline characteristics including age, sex, height, weight, and SMI among the groups (Table 1).

**TABLE 1:** Participants’ baseline characteristics.

	Placebo (*n* = 16)	Low-QG (*n* = 16)	High-QG (*n* = 16)	*P-value*
Age (y)^a^	59.8 ± 6.1	60.7 ± 6.1	60.1 ± 7.8	0.930
Sex (men/women)^b^	8/8	7/9	7/9	0.920
Height (cm)^a^	161.8 ± 6.1	161.6 ± 10.4	159.5 ± 11.2	0.754
Weight (kg)^a^	57.8 ± 8.4	58.5 ± 9.2	57.4 ± 12.9	0.952
SMI (kg/m^2^)^a^	7.0 ± 0.9	7.0 ± 1.1	7.0 ± 1.1	0.993

*Values are expressed as means ± standard deviation. There was no significant difference among the groups in baseline data (^a^ANOVA, ^b^chi-squared test). QG, quercetin glycoside; SMI, skeletal muscle quantity index.*

### Muscle Quantity and Stiffness in the Per-Protocol Set Analysis

**FIGURE 2:**
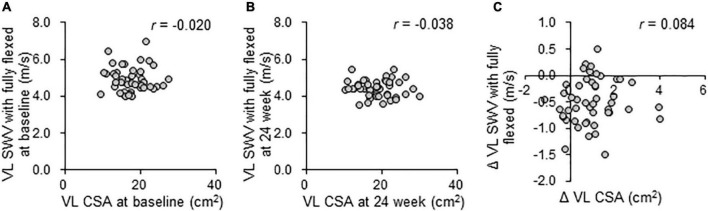
Relationships between muscle quantity and stiffness during the 24-week intervention in the PPS analysis. Pearson’s correlation coefficient (*r*) between VL muscle CSA on MRI and SWV with the knee fully flexed on SWE at baseline **(A)** and at 24 weeks **(B)**, and the changes during the 24-week intervention **(C)** in the PPS analysis. VL, vastus lateralis; CSA, cross-sectional area; and SWV, shear wave velocity.

The combined effects of QG supplementation and resistance training during the 24-week intervention on muscle quantity and stiffness were evaluated using MRI, DXA, and SWE measurements (Table 2). There were no significant differences among the groups in the parameters at baseline. During the 24-week intervention period, a significant group × time interaction was not observed in thigh muscle CSA set as the primary outcome, as well as the VL muscle CSA and lean body mass measured by MRI and DXA. Regarding VL SWV, there was a significant group × time interaction for the knee fully flexed position (*p* = 0.023), but no significant interaction was observed for the knee fully extended and knee flexed at 90° positions. VL SWV in the knee fully flexed position at 24 weeks was significantly decreased compared with baseline (–0.3 m/s in the placebo group, *p* < 0.01; –0.6 m/s in the low-QG group, *p* < 0.01; –0.6 m/s in the high-QG group, *p* < 0.01). The changes in SWV in the knee fully flexed position over 24 weeks were significantly larger in both the low and high-QG groups than in the placebo group (low-QG vs. placebo: *p* < 0.05, high-QG vs. placebo: *p* < 0.05). A correlation between VL muscle CSA and SWV measured in the knee fully flexed position was not observed at baseline (*r* = –0.020; *p* = 0.893, Figure 2A) and at 24 weeks (*r* = –0.038; *p* = 0.800, Figure 2B). Moreover, there was no correlation between the changes in SWV and VL muscle CSA during the 24-week intervention (*r* = 0.084; *p* = 0.571, Figure 2C). No correlation was also observed between VL SWV and 1-RM muscular strength (Supplementary Figure 1). 1-RM of all training was increased in a time-dependent manner in the three groups, but there was no significant group × time interaction (Supplementary Table 1).

**TABLE 2:** Effects of the intervention on muscle quantity and stiffness in the PPS analysis.

Variable	Group	Baseline	12 weeks	24 weeks	Change (Δ 12 weeks)	Change (Δ 24 weeks)	Two-way ANOVA (group × time) *P-value*
**MRI measurements**							
Thigh muscle CSA (cm^2^)	Placebo	98.7 ± 17.4	102.0 ± 18.5	104.2 ± 19.2	3.3 ± 2.6	5.5 ± 3.6	0.915
	Low-QG	102.7 ± 25.4	106.2 ± 25.8	109.0 ± 28.2	3.4 ± 2.4	6.3 ± 4.4	
	High-QG	101.0 ± 25.3	105.1 ± 26.9	107.3 ± 27.4	4.1 ± 2.6	6.3 ± 4.5	
VL muscle CSA (cm^2^)	Placebo	17.0 ± 3.6	17.6 ± 3.8	17.8 ± 3.9	0.6 ± 0.7	0.9 ± 0.9	0.635
	Low-QG	18.3 ± 4.1	18.9 ± 4.2	19.5 ± 5.0	0.6 ± 0.8	1.2 ± 1.3	
	High-QG	17.7 ± 4.6	18.1 ± 4.6	18.5 ± 4.9	0.5 ± 0.7	0.8 ± 0.9	
**DXA measurements**							
Leg lean mass (kg)	Placebo	13.2 ± 2.3	13.5 ± 2.5	13.6 ± 2.7	0.3 ± 0.4	0.3 ± 0.6	0.332
	Low-QG	13.7 ± 3.8	14.1 ± 3.9	13.9 ± 3.9	0.5 ± 0.4	0.2 ± 0.4	
	High-QG	13.1 ± 3.4	13.3 ± 3.6	13.4 ± 3.5	0.2 ± 0.5	0.3 ± 0.3	
Arm lean mass (kg)	Placebo	4.1 ± 1.1	4.2 ± 1.1	4.2 ± 1.1	0.1 ± 0.1	0.0 ± 0.1	0.489
	Low-QG	4.2 ± 1.4	4.2 ± 1.4	4.2 ± 1.4	0.0 ± 0.1	0.0 ± 0.2	
	High-QG	4.1 ± 1.3	4.1 ± 1.3	4.1 ± 1.2	0.1 ± 0.2	0.0 ± 0.1	
Whole-body lean mass (kg)	Placebo	40.0 ± 6.9	40.7 ± 6.9	40.8 ± 7.3	0.6 ± 0.6	0.8 ± 0.9	0.904
	Low-QG	40.6 ± 9.9	41.4 ± 10.1	41.4 ± 10.3	0.7 ± 0.7	0.8 ± 1.0	
	High-QG	39.8 ± 9.4	40.3 ± 9.4	40.6 ± 9.1	0.5 ± 0.6	0.8 ± 1.0	
**SWE measurements of VL**							
SWV with the knee fully extended (m/s)	Placebo	2.0 ± 0.1	2.0 ± 0.1	2.0 ± 0.1	0.0 ± 0.1	0.0 ± 0.1	0.452
	Low-QG	1.9 ± 0.1	1.9 ± 0.2	1.9 ± 0.1	0.0 ± 0.2	0.0 ± 0.1	
	High-QG	2.0 ± 0.1	1.9 ± 0.2	2.0 ± 0.1	0.0 ± 0.1	0.0 ± 0.1	
SWV with the knee flexed at 90° (m/s)	Placebo	3.0 ± 0.2	2.9 ± 0.2	2.8 ± 0.2	–0.1 ± 0.2	–0.2 ± 0.2	0.811
	Low-QG	2.9 ± 0.2	2.8 ± 0.3	2.7 ± 0.1	–0.1 ± 0.2	–0.1 ± 0.2	
	High-QG	2.9 ± 0.3	2.8 ± 0.3	2.8 ± 0.2	–0.2 ± 0.2	–0.2 ± 0.2	
SWV with the knee fully flexed (m/s)	Placebo	4.7 ± 0.6	4.6 ± 0.5	4.4 ± 0.4^§§^	–0.1 ± 0.3	–0.3 ± 0.4	0.023
	Low-QG	5.0 ± 0.5	4.7 ± 0.6^§§^	4.4 ± 0.4^§§^	–0.4 ± 0.3	–0.6 ± 0.3*	
	High-QG	5.0 ± 0.8	4.8 ± 0.8	4.4 ± 0.5^§§^	–0.2 ± 0.5	–0.6 ± 0.5*	

Values are expressed as means ± standard deviation. For the placebo (n = 16), low-QG (n = 16), and high-QG (n = 16) groups on MRI, DXA, and SWE measurements, where one set of SWE measurements in the placebo group at 12 weeks was missing because of no visit, there were no significant differences among the groups at baseline (one-way ANOVA). *p < 0.05 compared with placebo group (Dunnett’s test). ^§§^p < 0.01 compared with values at baseline (Dunnett’s test). QG, quercetin glycoside; MRI, magnetic resonance imaging; CSA, cross-sectional area; VL, vastus lateralis; DXA, dual energy X-ray absorptiometry; SWE, shear wave elastography; and SWV, shear wave velocity.

### Safety

No side effects due to QG supplementation were observed. There were no severe adverse events and no significant difference in the incidence of adverse events among the placebo (70.6%), low-QG (52.9%), and high-QG (70.6%) groups.

## Discussion

In the present study, the 24-week intervention effects of QG supplementation combined with low-intensity resistance training on muscle quantity and stiffness (as an index of fibrosis) were investigated in middle-aged and elderly people. For muscle quantity, there were no significant differences in thigh CSA on MRI between QG supplementation with exercise and placebo with exercise, as well as each lean body mass on DXA. For muscle stiffness, both 200 and 500 mg QG supplementation with exercise significantly decreased SWV of VL in a stretched position (i.e., knee fully flexed) compared to placebo with exercise. This is the first report to show that the combination of nutrition and exercise could improve age-related changes in passive muscle stiffness.

Extracellular matrix components are responsible for muscle stiffness (3, 20). In human, stiffness of muscle bundles, which consist of several muscle fibers and contain ECM components, is more than 15 times higher than that of single muscle fibers, although the muscle bundle cross-sectional area contains only 5% of ECM components (21). Thus, it is reasonable to use muscle stiffness as an index of ECM components. Previously, we identified an increase of VL muscle stiffness in full knee flexion from 47.9 years of age, indicating that the ECM components in skeletal muscle increased as one of the age-related changes in muscle quality (7). In animal studies, the excessive ECM accumulation in older muscles was reduced by resistance training with change of the gene expression related to ECM turnover (22, 23). Moreover, the acute stimulus of resistance exercise also decreased the gene expression associated with skeletal muscle ECM remodeling in elderly men (24). However, no longitudinal studies evaluating the effects of exercise on muscle fibrosis caused by aging have been available. The present study is the first to demonstrate the effects of long-term resistance training on ECM accumulation in middle-aged and elderly people (Table 2), albeit indirectly through muscle stiffness assessment using ultrasound SWE. Furthermore, QG supplementation with both 200 and 500 mg combined with exercise improved muscle stiffness more than exercise alone (Table 2). To support these data, administration of quercetin, an active form of QGs in target tissues, decreased muscle fibrosis by suppressing inflammatory cytokines in mdx mice (18). In addition, no significant correlation was observed between muscle stiffness in the stretched position and muscle CSA in VL, as well as their changes during the 24-week intervention (Figure 2), indicating that the intervention effects on muscle stiffness were independent of those on muscle quantity. These findings suggest that QG supplementation exerts additive effects on the improvement of passive muscle stiffness with resistance training. Moreover, the effects of various interventions and age-associated differences in muscle stiffness were observed only in stretched positions, but not in shortened positions in the previous studies (7, 25, 26). This was consistent with the results of the present study. Muscle stiffness in stretched positions can reflect the accumulation of ECM components referred to as muscle fibrosis (7). Thus, the present findings indicate that a combination of exercise and QG supplementation improves age-associated muscle fibrosis. The present results showed no correlations between muscle stiffness and 1-RM muscular strength (Supplementary Figure 1), suggesting that muscle stiffness would relate to muscle function other than muscular strength. Based on the facts that increased muscle stiffness impairs joint flexibility (4, 27) and that a decline in joint flexibility impairs balance and functional ability (28), decreasing the quality of life for elderly people, the reduction in muscle stiffness by exercise and QG supplementation observed in the present study would contribute to increased joint flexibility, and then improve the quality of life in elderly people, although further studies are needed to clarify this hypothesis.

In terms of the effects of QG supplementation on muscle quantity, PPS analysis showed no significant group × time interaction in muscle CSAs and lean mass (Table 2), suggesting that combined effects of QG supplementation and exercise on muscle quantity were not observed in the present study. Previous studies in animals indicated that quercetin, an active form of QGs, prevented muscle atrophy by suppressing ubiquitin proteasome system-induced muscle degradation in response to several factors, such as disuse, glucocorticoids, obesity, and inflammation (16, 17, 29, 30). In general, muscle mass is regulated by the balance of muscle synthesis and degradation, and several factors such as aging and inactivity enhance muscle degradation to reduce muscle mass (31). Thus, it was assumed that the effects of QG supplementation on muscle mass might be confirmed in subjects who were slightly losing muscle mass, combined with exercise which increases muscle synthesis. Actually, participants in the present study were 50–74 years old, whose average SMI (7.9 kg/m^2^ in men, 6.2 kg/m^2^ in women) was relatively higher than the cut-off value of sarcopenia in the Asian Working Group for Sarcopenia in 2019 (7.0 kg/m^2^ in men, 5.7 kg/m^2^ in women; 11). Thus, we additionally conducted the subgroup analysis of the low SMI group, whose SMIs were lower than each average of men and women, respectively (Supplementary Table 2). According to the PPS analysis, significant changes were observed in muscle stiffness (Supplementary Table 3), and there were no correlations of muscle stiffness with quantity or muscular strength (Supplementary Figure 2). There were also no significant group × time interaction for muscle quality, but the changes of whole-body lean mass by QG supplementation with both 200 mg (+0.6 kg, *p* < 0.05 vs. baseline) and 500 mg (+1.2 kg, *p* < 0.01 vs. baseline) combined with exercise were higher than those with exercise alone (+0.3 kg, *p* > 0.05 vs. baseline) during the 24-week intervention (Supplementary Table 3) if conducting the statistical analysis for comparing before and after the intervention in each group. Of note, the effects of QG supplementation on muscle quantity was observed only in whole-body lean mass on DXA, not in thigh muscle CSA on MRI (Supplementary Table 3). Our previous report showed that muscle CSAs on MRI would be more sensitive for detecting local muscle hypertrophy by resistance training than lean mass on DXA (10), which was confirmed in the present study; the changes in thigh muscle CSA (+5.6%) were higher than those of whole-body lean mass (+2.0%) by exercise alone during the 24-week intervention. In contrast, nutritional interventions may exert systemic effects on skeletal muscle, so that lean mass on DXA would be more appropriate for evaluating these effects. In fact, many studies have shown the effects of protein supplementation combined with exercise on muscle mass using DXA (32), and it was reported that increased dietary protein intake enhanced whole-body lean mass, but not thigh muscle CSA, in a 10-week intervention for elderly men (33). Nonetheless, it may be well worth noting that the increase in whole-body lean mass could be elicited by the addition of QG supplementation to even low-intensity resistance exercise, at least in individuals with lower SMIs. Confirmatory studies with a larger sample size are needed to clarify the efficacy of QG supplementation on muscle quantity.

Most previous studies used 1,000 mg of quercetin aglycone per day for oral administration to evaluate its physiological effects in clinical studies (34, 35). The QGs used in the present study are more water-soluble and have higher bioavailability, over 10-fold compared to quercetin aglycone (36). Regarding the difference in absorption capacity, over 200 mg of QG may be needed to exert physiological actions similar to 1,000 mg of quercetin aglycone. Of note, a recent study demonstrated increased neuromuscular efficacy by a single ingestion of 500 mg QGs (37). The present study showed that the combined effects of 200 mg of QGs with low-intensity resistance exercise on muscle quantity and quality are similar to those of 500 mg of QGs. Therefore, supplementation with 200 mg QGs would be enough to obtain sufficient combined effects with resistance training, at least in muscle quantity and quality, which may be physiologically relevant in terms of safety and feasibility as a supplement.

There were some limitations in the present study. First, the evaluation used by the MRI technique was only for muscle CSAs at midthigh, but not muscle volume. The relative increase in the muscle cross-sectional area (i.e., the magnitude of muscle hypertrophy) is non-uniform along its length, as reported by previous studies (38, 39). However, based on the previous findings, the CSA change at the central site is almost the average of overall changes. Additionally, it is unlikely that muscle belly length is substantially increased by resistance training. Thus, we believe that the change in muscle CSA at the central site can represent that in muscle volume. Secondly, the estimative of 1-RM performance by the indirect method of 1-RM test would be likely underestimated in the elderly people (40). However, training effect (i.e., the pre-to-post change) might not be affected, because the evaluation by the well-trained instructor was common at each time point. We need to consider the use of direct 1-RM test for future studies to clarify the relationships between changes of muscle stiffness and performance. Conversely, the present study had some strengths. The participants’ compliance with exercise intervention and capsule intake was very high. Moreover, there were no severe adverse events by both QG supplementation and exercise and no significant difference in the incidence of adverse events among groups. Therefore, it was considered that exercise and QG supplementation were performed adequately based on the study design. Thus, it is reasonable to say that the results from the present study provided evidence that the combination of QG supplementation and exercise improved both muscle quantity and quality.

In conclusion, the combination of QG supplementation and low-intensity resistance training exercise improved the passive muscle stiffness (as an index of muscle fibrosis) induced by aging to a similar degree with QG supplementation of 200 and 500 mg. The present study demonstrates that a combination of exercise and nutrition would be useful for improving age-related changes in muscle quantity and quality, thus preventing sarcopenia in elderly people.

## Data Availability Statement

The original contributions presented in this study are included in the article/Supplementary Material, further inquiries can be directed to the corresponding author.

## Ethics Statement

The studies involving human participants were reviewed and approved by the Ethics Committee of The Fukuda Clinic and Ritsumeikan University. The patients/participants provided their written informed consent to participate in this study.

## Author Contributions

YO, NM, AN, TI, MN, TA, YY, and TH designed the study. YO, AN, TI, and MF conducted the clinical trial and were responsible for the exercise intervention and supplementation. NM, TA, YY, and TH were involved in the investigation of muscle quantity and quality. YO performed statistical analysis and data interpretation with guidance from NM, TA, YY, and TH. YO wrote the manuscript. NM, TI, and TH revised the manuscript. All authors have read and agreed to the published version of the manuscript.

## Conflict of Interest

This study received funding from Suntory Wellness Ltd. YO, AN, TI, and MN Were employees of Suntory Wellness Ltd., which manufactures and sells health food products. The funder had the following involvement with the study: the study design, collection, analysis, interpretation of data, the writing of this article, and the decision to submit it for publication. The remaining authors declare that the research was conducted in the absence of any commercial or financial relationships that could be construed as a potential conflict of interest.

## Publisher’s Note

All claims expressed in this article are solely those of the authors and do not necessarily represent those of their affiliated organizations, or those of the publisher, the editors and the reviewers. Any product that may be evaluated in this article, or claim that may be made by its manufacturer, is not guaranteed or endorsed by the publisher.

## Abbreviations

QG: quercetin glycoside
CSA: cross-sectional area
VL: vastus lateralis
1-RM: 1-repetition maximum
MRI: magnetic resonance imaging
DXA: dual-energy X-ray absorptiometry
SWE: shear wave elastography
SWV: shear wave velocity
SMI: skeletal muscle mass index
PPS: per-protocol set
ANOVA: analysis of variance.
