# Erratum: Effects of quercetin glycoside supplementation combined with low-intensity resistance training on muscle quantity and stiffness: A randomized, controlled trial

**DOI:** 10.3389/fnut.2022.1028810

**Published:** 2022-09-27

**Authors:** Frontiers Production Office

**Affiliations:** Frontiers Media SA, Lausanne, Switzerland

**Keywords:** muscle quality, shear wave elastography, passive muscle stiffness, whole-body lean mass, nutrition and exercise

Due to a production error, there was a mistake in Table 2 as published. In Table 2, the asterisk mark (^*^), indicating ^*^p < 0.05 compared with the placebo group (Dunnett's test), is incorrectly shown in the row “SWV with the knee fully flexed,” subcategory “Placebo,” and column “Change (**Δ** 24 weeks).” The corrected Table 2 appears below.

**Table 2:** Effects of the intervention on muscle quantity and stiffness in the PPS analysis.

**Variable**	**Group**	**Baseline**	**12 weeks**	**24 weeks**	**Change (Δ 12 weeks)**	**Change (Δ 24 weeks)**	**Two-way ANOVA (group × time) *P-value***
MRI measurements							
Thigh muscle CSA (cm^2^)	Placebo	98.7 ± 17.4	102.0 ± 18.5	104.2 ± 19.2	3.3 ± 2.6	5.5 ± 3.6	0.915
	Low-QG	102.7 ± 25.4	106.2 ± 25.8	109.0 ± 28.2	3.4 ± 2.4	6.3 ± 4.4	
	High-QG	101.0 ± 25.3	105.1 ± 26.9	107.3 ± 27.4	4.1 ± 2.6	6.3 ± 4.5	
VL muscle CSA (cm^2^)	Placebo	17.0 ± 3.6	17.6 ± 3.8	17.8 ± 3.9	0.6 ± 0.7	0.9 ± 0.9	0.635
	Low-QG	18.3 ± 4.1	18.9 ± 4.2	19.5 ± 5.0	0.6 ± 0.8	1.2 ± 1.3	
	High-QG	17.7 ± 4.6	18.1 ± 4.6	18.5 ± 4.9	0.5 ± 0.7	0.8 ± 0.9	
DXA measurements							
Leg lean mass (kg)	Placebo	13.2 ± 2.3	13.5 ± 2.5	13.6 ± 2.7	0.3 ± 0.4	0.3 ± 0.6	0.332
	Low-QG	13.7 ± 3.8	14.1 ± 3.9	13.9 ± 3.9	0.5 ± 0.4	0.2 ± 0.4	
	High-QG	13.1 ± 3.4	13.3 ± 3.6	13.4 ± 3.5	0.2 ± 0.5	0.3 ± 0.3	
Arm lean mass (kg)	Placebo	4.1 ± 1.1	4.2 ± 1.1	4.2 ± 1.1	0.1 ± 0.1	0.0 ± 0.1	0.489
	Low-QG	4.2 ± 1.4	4.2 ± 1.4	4.2 ± 1.4	0.0 ± 0.1	0.0 ± 0.2	
	High-QG	4.1 ± 1.3	4.1 ± 1.3	4.1 ± 1.2	0.1 ± 0.2	0.0 ± 0.1	
Whole-body lean mass (kg)	Placebo	40.0 ± 6.9	40.7 ± 6.9	40.8 ± 7.3	0.6 ± 0.6	0.8 ± 0.9	0.904
	Low-QG	40.6 ± 9.9	41.4 ± 10.1	41.4 ± 10.3	0.7 ± 0.7	0.8 ± 1.0	
	High-QG	39.8 ± 9.4	40.3 ± 9.4	40.6 ± 9.1	0.5 ± 0.6	0.8 ± 1.0	
SWE measurements of VL							
SWV with the knee fully extended (m/s)	Placebo	2.0 ± 0.1	2.0 ± 0.1	2.0 ± 0.1	0.0 ± 0.1	0.0 ± 0.1	0.452
	Low-QG	1.9 ± 0.1	1.9 ± 0.2	1.9 ± 0.1	0.0 ± 0.2	0.0 ± 0.1	
	High-QG	2.0 ± 0.1	1.9 ± 0.2	2.0 ± 0.1	0.0 ± 0.1	0.0 ± 0.1	
SWV with the knee flexed at 90° (m/s)	Placebo	3.0 ± 0.2	2.9 ± 0.2	2.8 ± 0.2	−0.1 ± 0.2	−0.2 ± 0.2	0.811
	Low-QG	2.9 ± 0.2	2.8 ± 0.3	2.7 ± 0.1	−0.1 ± 0.2	−0.1 ± 0.2	
	High-QG	2.9 ± 0.3	2.8 ± 0.3	2.8 ± 0.2	−0.2 ± 0.2	−0.2 ± 0.2	
SWV with the knee fully flexed (m/s)	Placebo	4.7 ± 0.6	4.6 ± 0.5	4.4 ± 0.4^§§^	−0.1 ± 0.3	−0.3 ± 0.4	0.023
	Low-QG	5.0 ± 0.5	4.7 ± 0.6^§§^	4.4 ± 0.4^§§^	−0.4 ± 0.3	−0.6 ± 0.3*	
	High-QG	5.0 ± 0.8	4.8 ± 0.8	4.4 ± 0.5^§§^	−0.2 ± 0.5	−0.6 ± 0.5*	

Values are expressed as means ± standard deviation. For the placebo (n = 16), low-QG (n = 16), and high-QG (n = 16) groups on MRI, DXA, and SWE measurements, where one set of SWE measurements in the placebo group at 12 weeks was missing because of no visit, there were no significant differences among the groups at baseline (one-way ANOVA). ^*^p < 0.05 compared with placebo group (Dunnett's test). ^§§^p < 0.01 compared with values at baseline (Dunnett's test). QG, quercetin glycoside; MRI, magnetic resonance imaging; CSA, cross-sectional area; VL, vastus lateralis; DXA, dual energy X-ray absorptiometry; SWE, shear wave elastography; and SWV, shear wave velocity.

The publisher apologizes for this mistake. The original article has been updated.

