# Supplementary material for: Effects of Quercetin Glycoside Supplementation Combined With Low-Intensity Resistance Training on Muscle Quantity and Stiffness: A Randomized, Controlled Trial
Source: Front Nutr. 2022 Jul 6;9:912217. doi: 10.3389/fnut.2022.912217 (PMC9298516; doi:10.3389/fnut.2022.912217)
Supplement: Supplementary file 1 [file Data_Sheet_1.docx]

Supplementary Material

**Supplementary Tables**

Supplementary Table 1. Effects of the intervention on 1-RM in the PPS analysis

| Variable | Group |  |  |  |  | |  |  |  |  |
| --- | --- | --- | --- | --- | --- | --- | --- | --- | --- | --- |
| Leg extension  (kg) |  | Values at each time point | | | | | | | | Two-way ANOVA  (group*time) p-value |
|  |  | Baseline | 4 weeks | 8 weeks | 12 weeks | | 16 weeks | 20 weeks | 24 weeks |  |
|  | Placebo | 50.3 ± 13.7 | 62.0 ± 17.0 | 66.9 ± 17.4 | 70.5 ± 18.0 | | 74.3 ± 19.0 | 77.1 ± 19.4 | 80.2 ± 19.5 | 0.413 |
|  | Low-QG | 50.0 ± 16.0 | 66.8 ± 17.6 | 70.8 ± 19.1 | 74.2 ± 20.6 | | 80.3 ± 21.6 | 81.5 ± 23.0 | 82.2 ± 23.8 |  |
|  | High-QG | 49.5 ± 15.5 | 61.4 ± 17.4 | 65.8 ± 18.8 | 69.9 ± 20.3 | | 72.7 ± 22.1 | 74.3 ± 23.1 | 73.1 ± 20.7 |  |
|  |  | Changes from baseline | | | | | | | |  |
|  |  |  | Δ 4 weeks | Δ 8 weeks | Δ 12 weeks | | Δ 16 weeks | Δ 20 weeks | Δ 24 weeks |  |
|  | Placebo |  | 11.8 ± 7.5 | 16.6 ± 8.8 | 20.3 ± 9.5 | | 24.0 ± 10.4 | 26.9 ± 10.5 | 28.6 ± 11.1 |  |
|  | Low-QG |  | 16.8 ± 8.9 | 20.8 ± 10.3 | 23.3 ± 11.2 | | 29.9 ± 13.1 | 31.5 ± 14.5 | 32.2 ± 15.0 |  |
|  | High-QG |  | 11.8 ± 7.1 | 16.3 ± 8.9 | 19.1 ± 10.5 | | 23.1 ± 11.3 | 24.7 ± 12.2 | 26.0 ± 12.5 |  |
| Leg curl  (kg) |  | Values at each time point | | | | | | | | Two-way ANOVA  (group*time) p-value |
|  |  | Baseline | 4 weeks | 8 weeks | 12 weeks | | 16 weeks | 20 weeks | 24 weeks |  |
|  | Placebo | 26.4 ± 6.0 | 31.6 ± 7.2 | 33.4 ± 6.9 | 34.7 ± 6.5 | | 36.4 ± 7.2 | 37.0 ± 7.5 | 38.0 ± 7.9 | 0.923 |
|  | Low-QG | 27.2 ± 9.0 | 32.4 ± 10.0 | 34.4 ± 10.6 | 36.0 ± 11.1 | | 38.3 ± 11.8 | 39.3 ± 12.2 | 39.4 ± 12.3 |  |
|  | High-QG | 24.3 ± 10.6 | 29.1 ± 10.9 | 30.7 ± 11.4 | 32.4 ± 12.0 | | 33.7 ± 12.6 | 35.0 ± 13.4 | 33.9 ± 11.8 |  |
|  |  | Changes from baseline | | | | | | | |  |
|  |  |  | Δ 4 weeks | Δ 8 weeks | Δ 12 weeks | | Δ 16 weeks | Δ 20 weeks | Δ 24 weeks |  |
|  | Placebo |  | 5.3 ± 2.5 | 7.0 ± 2.9 | 8.4 ± 2.7 | | 10.0 ± 3.3 | 10.6 ± 3.6 | 11.3 ± 3.5 |  |
|  | Low-QG |  | 5.1 ± 5.0 | 7.2 ± 7.0 | 8.2 ± 5.1 | | 10.9 ± 7.5 | 12.0 ± 7.7 | 12.3 ± 7.9 |  |
|  | High-QG |  | 4.8 ± 2.4 | 6.4 ± 2.9 | 7.7 ± 2.8 | | 9.4 ± 3.3 | 10.6 ± 4.0 | 11.1 ± 4.1 |  |
| Leg press  (kg) |  | Values at each time point | | | | | | | | Two-way ANOVA  (group*time) p-value |
|  |  | Baseline | 4 weeks | 8 weeks | 12 weeks | | 16 weeks | 20 weeks | 24 weeks |  |
|  | Placebo | 142.1 ± 34.2 | 170.1 ± 45.3 | 182.6 ± 49.0 | 190.9 ± 49.6 | | 201.0 ± 53.2 | 206.9 ± 57.8 | 218.9 ± 58.8 | 0.448 |
|  | Low-QG | 144.8 ± 45.5 | 183.2 ± 52.4 | 194.4 ± 57.0 | 200.5 ± 60.4 | | 212.2 ± 59.0 | 215.9 ± 60.0 | 220.0 ± 64.2 |  |
|  | High-QG | 138.8 ± 44.3 | 166.3 ± 53.4 | 176.2 ± 55.6 | 188.2 ± 55.0 | | 189.3 ± 55.4 | 189.7 ± 53.3 | 193.5 ± 53.7 |  |
|  |  | Changes from baseline | | | | | | | |  |
|  |  |  | Δ 4 weeks | Δ 8 weeks | Δ 12 weeks | | Δ 16 weeks | Δ 20 weeks | Δ 24 weeks |  |
|  | Placebo |  | 28.0 ± 15.6 | 40.5 ± 20.5 | 48.4 ± 19.6 | | 59.0 ± 25.2 | 64.8 ± 29.8 | 74.2 ± 27.9 |  |
|  | Low-QG |  | 38.4 ± 23.3 | 49.6 ± 27.5 | 55.3 ± 32.3 | | 65.9 ± 32.7 | 71.0 ± 34.7 | 75.8 ± 37.4 |  |
|  | High-QG |  | 27.5 ± 20.8 | 37.4 ± 21.4 | 45.7 ± 24.2 | | 50.5 ± 27.9 | 55.8 ± 26.1 | 59.5 ± 27.1 |  |
| Chest press  (kg) |  | Values at each time point | | | | | | | | Two-way ANOVA  (group*time) p-value |
|  |  | Baseline | 4 weeks | 8 weeks | 12 weeks | | 16 weeks | 20 weeks | 24 weeks |  |
|  | Placebo | 42.0 ± 15.5 | 51.2 ± 16.6 | 53.1 ± 17.3 | 56.1 ± 18.4 | | 58.7 ± 19.6 | 59.2 ± 20.1 | 60.8 ± 19.9 | 0.837 |
|  | Low-QG | 40.1 ± 15.1 | 51.3 ± 17.9 | 54.2 ± 19.0 | 56.2 ± 20.4 | | 59.4 ± 20.4 | 60.1 ± 20.6 | 60.2 ± 21.3 |  |
|  | High-QG | 39.7 ± 15.6 | 47.7 ± 19.5 | 50.6 ± 19.8 | 54.8 ± 19.7 | | 55.4 ± 21.5 | 57.4 ± 21.8 | 55.3 ± 20.0 |  |
|  |  | Changes from baseline | | | |  | | | |  |
|  |  |  | Δ 4 weeks | Δ 8 weeks | Δ 12 weeks | | Δ 16 weeks | Δ 20 weeks | Δ 24 weeks |  |
|  | Placebo |  | 9.2 ± 4.6 | 11.1 ± 5.8 | 14.1 ± 6.6 | | 16.7 ± 7.6 | 17.3 ± 7.7 | 18.8 ± 7.7 |  |
|  | Low-QG |  | 11.2 ± 7.8 | 14.1 ± 9.3 | 16.0 ± 10.0 | | 18.7 ± 10.6 | 20.0 ± 10.7 | 20.8 ± 11.6 |  |
|  | High-QG |  | 7.9 ± 6.1 | 10.9 ± 6.8 | 13.8 ± 6.3 | | 15.6 ± 7.9 | 17.7 ± 8.1 | 16.8 ± 7.6 |  |

Values are expressed as mean ± standard deviation. For the placebo (*n* = 16), low-QG (*n* = 16), and high-QG (*n* = 16) groups on each 1-RM measurements, where 6 data (a 1-RM of leg press at 12 weeks, a 1-RM of leg extension, leg curl and two 1-RMs of leg press at 24 weeks in the placebo groups, and a 1-RM of leg press at 20 weeks in the high-QG group) were missing because of inadequate data, there were no significant differences among the groups at baseline (one-way ANOVA). QG, quercetin glycoside; 1-RM, 1-repetition maximum.

Supplementary Table 2. Baseline characteristics of participants in the subgroup with low SMI

|  | Placebo  (*n* = 8) | Low-QG  (*n* = 8) | High-QG  (*n* = 8) | *p*-value |
| --- | --- | --- | --- | --- |
| Age (years)^a^ | 60.0 ± 5.4 | 62.8 ± 4.7 | 64.4 ± 7.5 | 0.355 |
| Sex (men/women)^b^ | 5/3 | 3/5 | 3/5 | 0.511 |
| Height (cm)^a^ | 161.7 ± 6.2 | 160.3 ± 12.2 | 156.6 ± 12.8 | 0.629 |
| Weight (kg)^a^ | 54.1 ± 6.7 | 55.8 ± 9.1 | 53.1 ± 9.5 | 0.821 |
| SMI (kg/m^2^)^a^ | 6.8 ± 0.7 | 6.5 ± 1.1 | 6.3 ± 0.7 | 0.587 |

Values are expressed as mean ± standard deviation. There was no significant difference among the groups in baseline data (^a^ANOVA, ^b^chi-squared test). QG, quercetin glycoside; SMI, skeletal muscle mass index.

Supplementary Table 3. Effects of the intervention on muscle quantity and stiffness in the subgroup analysis by low SMI

| Variable | Group | Baseline | 12 weeks | 24 weeks | Change  (Δ 12 weeks) | Change  (Δ 24 weeks) | Two-way ANOVA  (group*time)  *p*-value |
| --- | --- | --- | --- | --- | --- | --- | --- |
| MRI measurements | | | | | | | |
| Thigh muscle CSA  (cm^2^) | Placebo | 89.7 ± 13.8 | 93.4 ± 14.3 | 94.9 ± 15.7 | 3.6 ± 1.8 | 5.1 ± 4.0 | 0.752 |
|  | Low-QG | 92.9 ± 22.3 | 96.0 ± 22.8 | 96.8 ± 23.6 | 3.1 ± 2.4 | 4.0 ± 2.6 |  |
|  | High-QG | 90.1 ± 22.1 | 94.5 ± 23.9 | 96.2 ± 25.8 | 4.5 ± 2.5 | 6.1 ± 5.3 |  |
| VL muscle CSA  (cm^2^) | Placebo | 15.2 ± 3.1 | 15.8 ± 3.5 | 16.0 ± 3.6 | 0.6 ± 0.9 | 0.8 ± 0.9 | 0.830 |
|  | Low-QG | 16.6 ± 4.1 | 17.4 ± 4.4 | 17.8 ± 5.1 | 0.8 ± 0.6 | 1.2 ± 1.3 |  |
|  | High-QG | 15.5 ± 4.0 | 16.0 ± 4.1 | 16.2 ± 4.6 | 0.5 ± 0.4 | 0.7 ± 1.0 |  |
| DXA measurements | | | | | | | |
| Leg lean mass  (kg) | Placebo | 12.6 ± 2.1 | 12.9 ± 2.2 | 12.8 ± 2.3 | 0.2 ± 0.3 | 0.2 ± 0.5 | 0.455 |
|  | Low-QG | 12.4 ± 3.8 | 12.9 ± 3.8 | 12.6 ± 3.8 | 0.4 ± 0.4 | 0.2 ± 0.5 |  |
|  | High-QG | 11.7 ± 3.1 | 11.9 ± 3.3 | 12.0 ± 3.1 | 0.2 ± 0.5 | 0.3 ± 0.2 |  |
| Arm lean mass  (kg) | Placebo | 4.0 ± 1.0 | 4.1 ± 1.0 | 4.0 ± 1.0 | 0.0 ± 0.1 | 0.0 ± 0.1 | 0.681 |
|  | Low-QG | 3.7 ± 1.4 | 3.7 ± 1.4 | 3.7 ± 1.3 | 0.0 ± 0.1 | 0.0 ± 0.2 |  |
|  | High-QG | 3.6 ± 1.2 | 3.7 ± 1.2 | 3.7 ± 1.2 | 0.1 ± 0.2 | 0.0 ± 0.1 |  |
| Whole-body lean  mass (kg) | Placebo | 39.0 ± 6.2 | 39.6 ± 6.2 | 39.3 ± 6.5 | 0.6 ± 0.7 | 0.3 ± 0.8 | 0.084 |
|  | Low-QG | 37.2 ± 9.7 | 38.0 ± 9.8 | 37.8 ± 9.6 | 0.8 ± 0.7 | 0.6 ± 0.9 |  |
|  | High-QG | 36.5 ± 8.9 | 37.0 ± 9.1 | 37.7 ± 9.0 | 0.6 ± 0.6 | 1.2 ± 0.9 |  |
| SWE measurements of VL | | | | | | | |
| SWV at the knee fully extended (m/s) | Placebo | 1.9 ± 0.1 | 2.0 ± 0.1 | 1.9 ± 0.1 | 0.1 ± 0.1 | 0.0 ± 0.1 | 0.944 |
|  | Low-QG | 2.0 ± 0.1 | 2.0 ± 0.3 | 1.9 ± 0.1 | 0.0 ± 0.2 | 0.0 ± 0.1 |  |
|  | High-QG | 2.0 ± 0.1 | 2.0 ± 0.1 | 2.0 ± 0.2 | 0.0 ± 0.2 | 0.0 ± 0.1 |  |
| SWV at the knee flexed at 90° (m/s) | Placebo | 3.0 ± 0.2 | 2.9 ± 0.2 | 2.8 ± 0.2 | ‐0.1 ± 0.2 | ‐0.2 ± 0.3 | 0.646 |
|  | Low-QG | 2.9 ± 0.3 | 2.9 ± 0.3 | 2.8 ± 0.2 | ‐0.1 ± 0.2 | ‐0.2 ± 0.2 |  |
|  | High-QG | 2.9 ± 0.4 | 2.7 ± 0.4 | 2.8 ± 0.3 | ‐0.2 ± 0.2 | ‐0.2 ± 0.2 |  |
| SWV at the knee fully flexed (m/s) | Placebo | 4.7 ± 0.6 | 4.6 ± 0.5 | 4.5 ± 0.4 | ‐0.1 ± 0.2 | ‐0.2 ± 0.3  *  ** | 0.003  * |
|  | Low-QG | 5.2 ± 0.4 | 4.7 ± 0.5^§§^ | 4.6 ± 0.4^§§^ | ‐0.4 ± 0.4 | ‐0.5 ± 0.2 |  |
|  | High-QG | 5.0 ± 0.8 | 4.7 ± 1.0^§^ | 4.2 ± 0.6^§§^ | ‐0.3 ± 0.4 | ‐0.8 ± 0.3 |  |

Values are expressed as mean ± standard deviation. For the placebo (*n* = 8), low-QG (*n* = 8), and high-QG (*n* = 8) groups on MRI, DXA, and SWE measurements, there were no significant differences among the groups at baseline (one-way ANOVA). **p* < 0.05 and ***p* < 0.01 compared with placebo group (Dunnett's test), ^§§^*p* < 0.01 compared with values at baseline (Dunnett's test). QG, quercetin glycoside; MRI, magnetic resonance imaging; CSA, cross-sectional area; VL, vastus lateralis; DXA, dual energy X-ray absorptiometry. SWE, shear wave elastography; SWV, shear wave velocity

Figure captions

Supplementary Figure 1. Relationships between 1-RM muscular strength and stiffness during the 24-week intervention in the PPS analysis

Pearson’s correlation co-efficient (*r*) between the sum of leg extension and leg press 1-RM (1-RM muscular strength) and SWV with the knee fully flexed on SWE at baseline (A) and at 24 weeks (B), and the changes during the 24-week intervention (C) in the PPS analysis. VL, vastus lateralis; CSA, cross-sectional area; 1-RM, 1-repetition maximum; SWV, shear wave velocity.

Supplementary Figure 2. Relationships between muscle quantity or 1-RM muscular strength and stiffness during the 24-week intervention in the subgroup analysis by low SMI.

Pearson’s correlation co-efficient (*r*) between VL muscle CSA on MRI or the sum of leg extension and leg press 1-RM (1-RM muscular strength) and SWV with the knee fully flexed on SWE at baseline (A, D) and at 24 weeks (B, E), and the changes during the 24-week intervention (C, F) in the subgroup analysis by low SMI. VL, vastus lateralis; SWV, shear wave velocity. 1-RM, 1-repetition maximum.
