# Supplementary figures and images for: Effects of Quercetin Glycoside Supplementation Combined With Low-Intensity Resistance Training on Muscle Quantity and Stiffness: A Randomized, Controlled Trial
Source: Front Nutr. 2022 Jul 6;9:912217. doi: 10.3389/fnut.2022.912217 (PMC9298516; doi:10.3389/fnut.2022.912217)

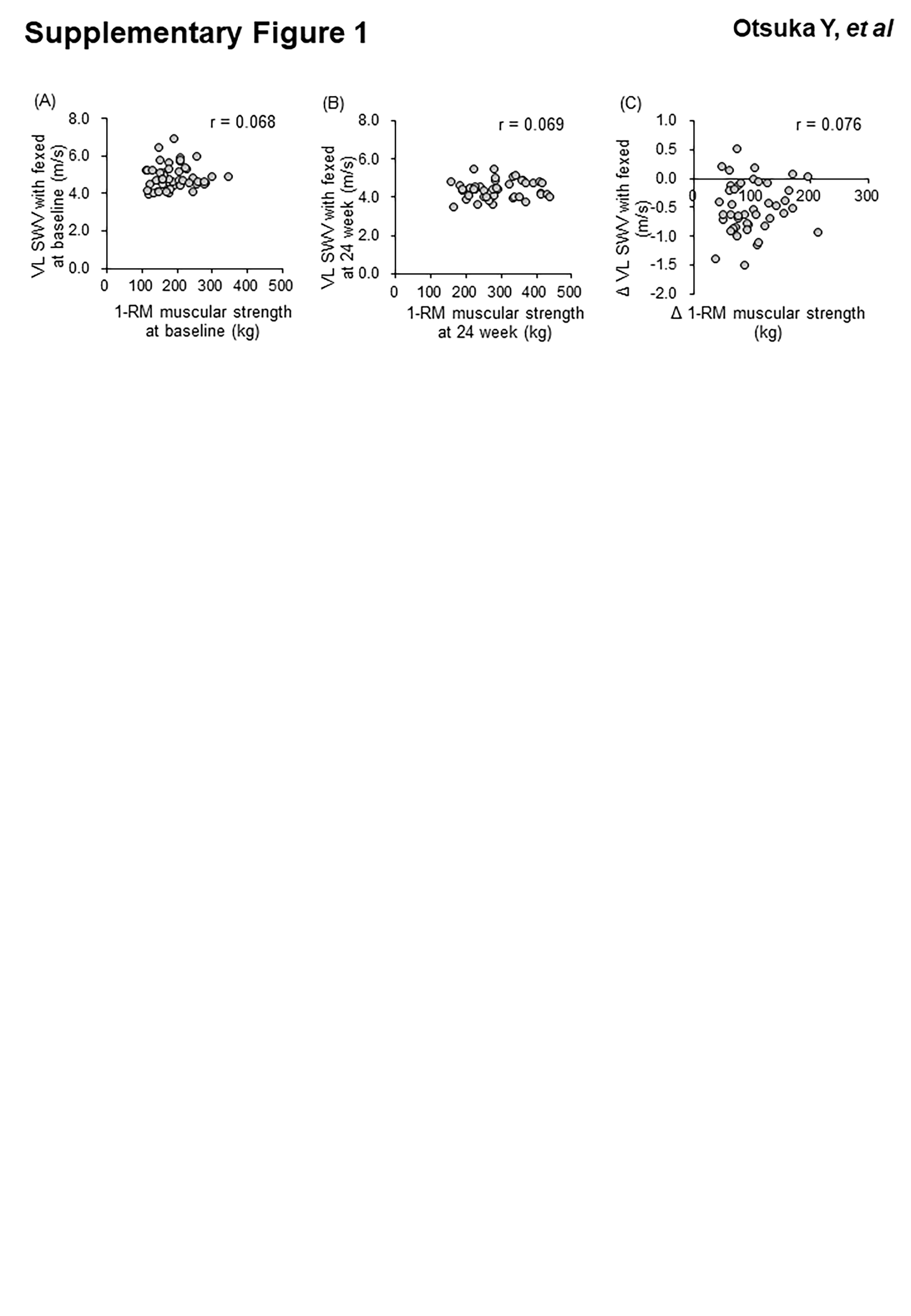

Supplement: Supplementary file 2 [file Image_1.TIF]

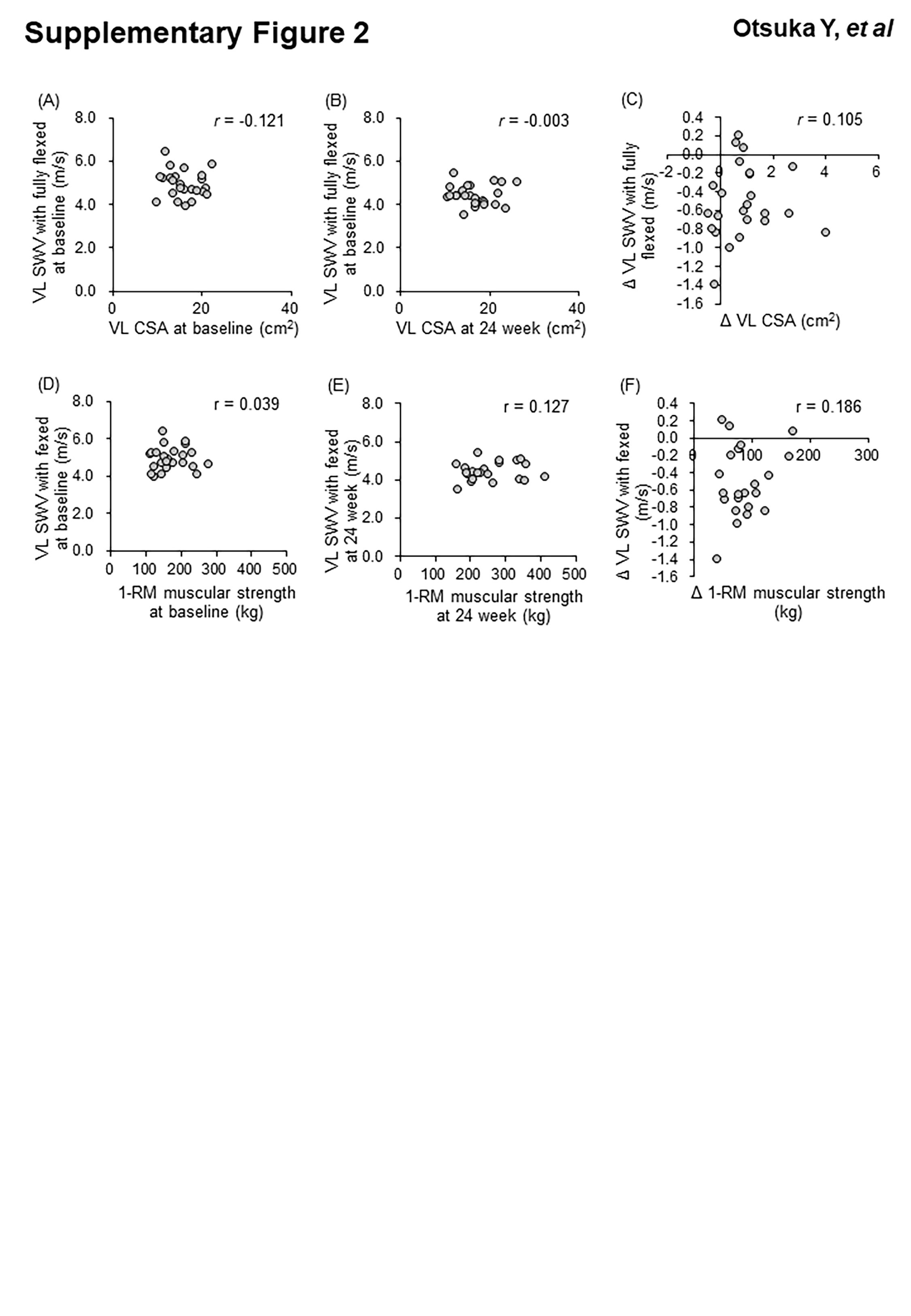

Supplement: Supplementary file 3 [file Image_2.TIF]
